# Cognitive Representation of Spontaneous Motion in a Second Language: An Exploration of Chinese Learners of English

**DOI:** 10.3389/fpsyg.2019.02706

**Published:** 2019-12-03

**Authors:** Yinglin Ji

**Affiliations:** School of Arts and Humanities, Shenzhen University, Shenzhen, China

**Keywords:** motion event typology, similarity judgment, linguistic relativity hypothesis, cognitive restructuring, spontaneous motion event

## Abstract

This study tests whether Chinese learners of English can reconstruct their cognitive pattern in the direction of the target system when judging the similarity between spontaneous motion screens in a match–to–sample task. English main verbs encode Manner of motion only, while Chinese verb compounds express Manner and Path simultaneously. Chinese monolinguals are thus predicted to develop a motion cognition pattern highlighting both Manner and Path salience whereas English monolinguals are more likely to be Manner-oriented. Our research findings are twofold. First, when assessed by the explicit measure of selection strategies (i.e., either Manner–match or Path-match), both monolingual and L2 learners show a general preference for the Path–match. However, when gauged by the implicit measure of processing speed (i.e., reaction time), Chinese monolinguals reacted significantly quicker than their English counterparts, particularly in making Path-matched judgments. Further, the L2 English learners across proficiencies responded significantly more slowly than their monolingual counterparts even at an advanced stage of acquisition, suggesting that the process of conceptual reconstructing, as demonstrated in our experiment, can be cognitively demanding and needs a longer period of time to complete. These findings are generally consistent with a weak version of the linguistic relativity hypothesis.

## Introduction

Human beings’ non–linguistic spatial understanding is believed to be universal because ‘our ability to perceive and interpret spatial relationships is supported by vision and other highly structured biological systems such as the haptic–kinesthetic system’ (Bowerman, 1999, p. 387). However, our linguistic systems exhibit striking variations in spatial description that do not reflect this perceptual and cognitive contour. This rich diversity in linguistic encoding of spatial events (motion in particular) has been widely documented in previous studies (Choi and Bowerman, 1991; Berman and Slobin, 1994; Slobin, 2004; Allen et al., 2007; Beavers et al., 2010; Ji et al., 2011a,b,c; Filipović and Jaszczolt, 2012, to name a few). It has been suggested that this effect of language can penetrate to the cognitive level, affecting how speakers of different languages conceptualize motion events in non-linguistic tasks (see, for instance, Slobin, 1996; Levinson, 2003; Hohenstein, 2005; Gullberg, 2011; Athanasopoulos and Bylund, 2013; Flecken et al., 2014; Ji and Hohenstein, 2017).

These observations have led to a revival of the linguistic relativity hypothesis in relation to space over the past three decades. However, the extension of the topic to the area of bilingualism is still a relatively new undertaking. The key questions asked include: if language can shape our thought pattern and different languages foster different thought patterns, what would happen to speakers who have command of more than one language? Will learning two languages affect motion cognition in a second language (L2) learner as a result of language–specific properties? Which factors, linguistic or social, contribute to the reconstruction of cognitive dispositions in L2 learners? In this context, the present study focuses on L2 motion event cognition in Chinese–English bilinguals (as compared to monolingual speakers) in a non–verbal similarity judgment task with the aim of revealing: (a) the extent to which the L2 English learners can change the thought pattern associated with their first language (L1) and adjust their cognitive propensity in the direction of an L2, and (b) the dynamic relationship between increasing proficiencies and stages of conceptual reconstructing in an L2 learner.

### Motion Event Typology and Its Cognitive Implications

In addition to Motion itself, Talmy (1985, 2000) conceptualizes motion events as consisting of Figure, Ground, Path and Manner. He further proposes a dichotomy between satellite–framed and verb–framed languages in motion description. In the former type of language, such as English and German, the main verb of a sentence typically conflates Manner of motion whilst expressing Path in verb particles (e.g*., The monkey climbed up a tree*); in the latter group of languages, which includes Spanish and Hebrew, the main verb of an utterance characteristically encodes Path (or direction) of motion. Therefore the default is for Manner information not to be expressed (e.g., *The monkey went up a tree* [*by climbing*]).

English has been widely accepted as a representative satellite–framed language whereas the typological picture of Mandarin Chinese (Chinese hereafter) is much more complicated. A motion event is typically expressed in Chinese through a verb compound, which usually consists of three parts: C1 (Manner verb) + C2 (Path element) + C3 (optional; Deictic element; e.g., *pa*2–*shang*4–*qu*4 ‘climb–ascend/up–go/thither’)^1^. Some researchers claim that Chinese can be taken as satellite–framed (Talmy, 1985, 2000) because C2 and C3 in the verb compound are akin to English particles: both belong to a closed–class set and are limited in number. Others, however, argue that it is more appropriate to consider Chinese as equipollenty-framed because C2 and C3 differ syntactically from English particles in frequently functioning as independent verbs (e.g., *Hou*2*zi shang*4 *le shu*4 ‘The monkey ascended the tree’ Gao, 2001; Slobin, 2004; Chen, 2005; Chen and Guo, 2009; Chu, 2009; Ji et al., 2011b).

In experimental studies concerning description of spontaneous motion events by Chinese adults and children, Ji et al. (2011a) found that even though we take C2 and C3 in Chinese verb compounds as ‘satellites’ and thus temporarily classify Chinese and English as both satellite–framed, about 25 per cent of spontaneous motion events in Chinese are expressed in quite different ways from English and show clear verb-framed properties (expressing Path alone in the main verb, e.g., *guo*4 ‘cross’ in example 1b).


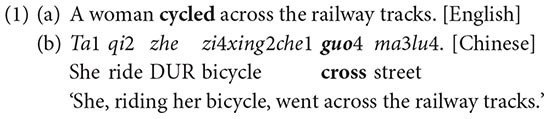


The linguistic differences between English and Chinese in motion description can have psychological and cognitive implications. In English, motion expressions involve syntactically unequal grammatical categories that must be matched with particular semantic components for Motion, i.e., Manner in verb and Path in verb particle. In Chinese, motion expressions include a resultative verb compound (RVC), which conveniently packages different semantic aspects of motion into one grammatical form. According to the linguistic relativity hypothesis, these language-specific properties affect a speaker’s ‘habitual behavior’, i.e., what speakers do most naturally, by default, in common situations. Although both Manner and Path are commonly encoded for motion expression in English and Chinese, the specific lexicalization patterns of motion events in the two languages prompt their speakers to pay differing amounts of attention to the varied semantic dimensions of motion. English speakers are systematically shown how to place Manner information in arguably the most important grammatical category of an utterance, i.e., the verb. Indeed, they habitually attend to Manner in verbs in their representation of motion events. In contrast, Chinese speakers systematically meet Manner coupled with Path in the verb and they habitually conceptualize these two types of information as being equally salient and as occurring simultaneously (see Ji, 2017, p. 68 for a more detailed discussion). According to the associative learning theory, “representations build up, or emerge, over exposure to a number of specific instances of associations” (Athanasopoulos et al., 2015, p. 141). Repeated exposures thus become part of an individual’s cognitive routine and can lead to regularities in conceptualization or behavior. Further, “the more routinized an association becomes, the easier it is to retrieve and utilize it for purposes of categorization” (Langacker, 2008; see Athanasopoulos et al., 2015, p. 141 for a detailed discussion).

### Motion Event Cognition in Monolingual and Bilingual Minds

The systematic differences in motion event description across languages have posed many questions for researchers, including whether the effect of motion event typology could affect how speakers of different languages conceptualize motion events. Such studies of non-linguistic cognition can directly address the linguistic relativity hypothesis with their focus on non–verbal processes, such as visual perception, triads matching and categorization.

For L1 speakers, techniques such as similarity judgments, eye–tracking and memory tests have produced some clear evidence for the linguistic relativity hypothesis when used in studies (Boroditsky, 2001; Levinson, 2003; Hohenstein, 2005; Ji and Hohenstein, 2018, to name a few). To cite an example, Hohenstein (2005) examined how differently aged English and Spanish children conceptualize spontaneous motion events. In the match-to-sample task using the ‘preferential looking’ scheme, she found that the participants’ behavior can be largely predicted by the typological properties of their L1s. The 7-year-old English speakers fixated on videos matching the Manner of a target video more often than Spanish-speaking 7-year-olds. The 3.5-year-olds in both language groups fixated on Path more often. Such findings provide powerful evidence for the linguistic relativity hypothesis: children’s fixation on motion scenes is similar prior to acquiring the spatial language patterns exhibited by adults (i.e., at the age of 3.5 years) but shows significant differences after such acquisitions (i.e., at the age of 7 years).

Although some studies report unclear or minimal effect of language on thought (Lucy, 1992; Gennari et al., 2002; Papafragou et al., 2002), they reveal that language-specific regularities made available in the experimental context can mediate the speaker’s performance in specific tasks. To illustrate, Gennari et al. (2002) investigated the effect of language processing on non-linguistic performance in recognition memory and similarity judgment tasks. Language-specific patterns were detected when participants were asked to orally describe motion scenes immediately prior to their participation in similarity judgment. This suggests that linguistic descriptions directed speakers’ attention to certain aspects of motion events later used to make a non-linguistic judgment.

L1 motion event conceptualization has been the focus of most research; fewer studies have systematically examined motion event cognition in an L2 context. However, in the past decade, there has been a growing interest in studies of thinking and speaking in two languages (Cadierno, 2004; Hohenstein et al., 2006; Athanasopoulos, 2009; Cook and Bassetti, 2011; Filipović, 2011; Pavlenko, 2011; Bylund and Athanasopoulos, 2014; Athanasopoulos et al., 2015; Brown, 2015; Montero-Melis et al., 2016; Thierry, 2016). These studies focus on processes that involve conceptual development and restructuring in bilingual speakers as a result of their additional language learning. Such research aims to determine linguistic variables (e.g., age of acquisition, language proficiency) as well as social factors (e.g., length of stay in target country, frequency of language use) that may affect bilingual cognition (see, Pavlenko, 2011, pp. 248–252 for a detailed discussion).

To give an example, a growing number of studies examine the relationship between grammatical aspect (e.g., perfect vs. imperfect) and the representation of motion events. Many studies have revealed that the presence or absence of grammatical aspect in a language influences the degree that speakers pay habitual attention to the endpoints of motion (see, for instance, Von Stutterheim et al., 2012; Athanasopoulos and Bylund, 2013; Bylund et al., 2013; Flecken et al., 2015). To illustrate, in one type of language (e.g., German or Swedish), the verb does not have aspectual inflections and the speakers of these non–aspect languages tend to focus on the endpoint of a motion event, thus developing a holistic perspective of event (e.g., *She is walking toward the church*). In contrast, verbs in some other languages are marked for aspect (e.g., the progressive in English) and users of these aspect languages are inclined to direct their attention to the ‘ongoingness’ of an event, thereby taking an ‘inside’ view of a situation (e.g., *She is walking along the road* [to the church]; see Flecken et al., 2015, pp. 42–43 for a summary). This correlation between grammatical aspect and event endpoints is attested in behavioral tasks, such as eye movements in viewing motion screens, and motion event categorization.

Extending this effect of language difference on visual perception of motion to the bilingual domain, the key question arises as to whether, and how, L2 learners can recalibrate their conceptual (or cognitive) propensity associated with L1 as a result of their additional language learning. Athanasopoulos et al. (2015) address this question in a triads matching task by examining how English (aspect language focusing on ‘ongoingness’ of an event) learners of German (non–aspect language highlighting endpoints of motion event) match a target scene with an intermediate degree of endpoint tendency (e.g., a person walking toward a car) to two alternates showing high vs. low degree of endpoint saliency (e.g., a person walking into a building vs. a person walking along a road, with a building in the distance). Their findings reveal that English learners of German become inclined to make their judgments on the basis of endpoint saliency (i.e., the value of L2) as their L2 proficiency increases and with longer exposure to the target language, thus suggesting a conceptual shift or reconstruction largely achieved.

Most studies of L2 motion cognition involve languages with opposing typological properties (e.g., satellite– vs. verb–framed). There are a number of studies exploring intratypological variations of motion expressions, but they seem to focus on lexicalization patterns of motion events and do not go beyond the linguistic level (see, for instance, Filipović, 2010, p. 263 for Serbo–Croatian, Kopecka, 2010, p. 237 for Polish, and Iakovleva, 2012 for Russian). In fact, very few studies have systematically explored the ‘degree of difference’ amongst languages in the same (or similar) typological category and the effect of such subtle linguistic differences on motion event cognition. One exception is the work of Czechowska and Ewert (2011), who argue that the group of satellite–framed languages is not homogeneous. For example, both English and Polish characteristically encode Manner in motion description, but the Path dimension has a higher degree of ‘codability’ in English than in Polish (i.e., English provides more accessible means of expressing Path as compared to Polish). They design similarity judgment and rating tasks to explore whether such minimal differences in motion lexicalization pattern have cognitive implications. Their results show that English monolinguals pay more attention to Path in the rating task than their Polish counterparts when their attention is directed to more than one attribute of motion at the same time. Furthermore, Polish–English bilinguals behave like monolingual speakers of English in their attention to Path. There is a linear relationship between L2 proficiency and perception of motion events, clearly suggesting a shift toward L2 values. Such findings lead to Czechowska and Ewert’s (2011) conclusion that a conceptual shift toward the L2 has already taken place in the least–proficient bilinguals and that reconstructing of the conceptual domain is evidenced in the two most proficient groups (2011, p. 308).

In a similar fashion, Ji (2017) investigated how bilinguals with typologically partially similar languages (satellite–framed English and equipollently–framed Chinese) behave in a triads matching experiment with motion stimuli illustrating a complicated type of caused motion event (e.g., *The boy rolling a ball down slope*). When gauged by implicit continuous measurement of reaction time (RT), the pattern of response latency in Chinese vs. English native speakers shows a clear effect of motion event typology in the first instance. English monolinguals react significantly more quickly in judging Manner–matched screens compared with Chinese monolinguals, who tend to make Manner– and Path–matched decisions in roughly the same amount of time. Furthermore, L2 English learners with intermediate to high proficiencies generally produce target–like responses, though the response pattern of L2 beginners still retains influence from L1 linguistic constraints. Such findings echo those of Czechowska and Ewert (2011), both suggesting that even subtle differences in lexicalization strategy in motion event description can have important cognitive implications for L2 learners as well as monolingual speakers.

To summarize, much ground has yet to be covered in studies of bilingual motion event cognition. Research into languages with minimal typological differences (rather than opposing typological features) is in great need of expansion. In this context, the present study aims to reveal whether, and how, the partial resemblance between English and Chinese in motion description can facilitate L2 learners’ conceptual switch or convergence toward the L2 value, as measured in non–verbal tasks.

### Predictions

Due to language–specific properties, we hypothesize, first of all, that Chinese monolinguals will be Manner–and–Path oriented in the behavioral task whereas their English counterparts will be predominately Manner–oriented. We speculate that the L2 Chinese learners of English at the initial stage of their acquisition will resemble Chinese monolinguals more closely and remain largely Manner–and–Path oriented. Only when L2 learners progress to higher levels of learning (e.g., at intermediate and advanced levels) will their behavior become target–language–like and become largely Manner–oriented. This prediction is made after taking into account two arguments:

(a) According to Slobin’s (1996, p. 89) ‘thinking for speaking’ hypothesis, learning a second language means acquiring an alternative way of thinking. Therefore, the L1 ‘thinking for speaking’ pattern, which is ingrained from one’s childhood, should be resistant to reconstruction in adult second language learning. We speculate that this is particularly true at an early stage of acquisition.
(b) Previous studies suggest that learning a second language involves changing one’s existing concepts or developing new concepts (see, for instance, Pavlenko, 2011). We argue that this process needs to be completed and internalized over a longer period of time. Developmental changes in behavior (if any) thus only occur at a later stage of acquisition.

Specifically, we predict two possibilities regarding the explicit measure of selection strategies and the implicit measure of processing time:

(a) A strong version of the linguistic relativity hypothesis: a subtle effect of language on cognition is manifested in both overt choices and response latencies. Chinese monolinguals, as well as low proficiency L2 learners, will choose the Path–matched videos as most similar to the target video more often than English native speakers and relatively advanced L2 learners. Meanwhile, they will show more rapid processing of the Path dimension, as evidenced by their significantly shorter RT in judging videos with Path–similarity, presumably due to the higher linguistic codability of Path in the Chinese language.
(b) A weak version of the linguistic relativity hypothesis: variations in the thought pattern as produced by linguistic differences will only be attested at the automatic and implicit level of processing (i.e., the RT), but not at the explicit level of forced judgments (i.e., A or B choices). Following this argument, we expect Chinese monolinguals and low proficiency L2 learners of English to react significantly more quickly in judging Path–matched (rather than Manner–matched) videos, compared with English monolinguals and advanced L2 learners.

In terms of preferences in judgment, however, we predict that participants across groups will mainly choose Path–matched scenes. This prediction is based on the following observations:

(a) The two languages under investigation in the present study do not have opposing typological properties in motion description (English and Chinese are partially similar), and this lesser magnitude of linguistic difference may not be strong enough to elicit different cognitive modes in behavioral tasks (see Lupyan, 2012 for a detailed discussion).
(b) Path of motion (rather than Manner of motion) has cognitive salience in the conceptualization of motion events (Talmy, 1985; Ibarretxe-Antuñano, 2009). It is the most essential motion component, without which a motion event hardly exists.

Note that the prediction of a possible weak version of the linguistic relativity hypothesis is also supported by findings from some previous studies, which suggest that there is usually a divergence between explicit measure (e.g., decision strategies in the present study) and implicit measure (i.e., RT) of second language learning (see, for instance, Li et al., 1993; Tokowicz and MacWhinney, 2005).

## Methodology

### Participants

A total of 160 students participated in this study. They were divided into 5 groups with 32 participants per group (16 females and 16 males). The monolingual speakers of English were recruited through a university in London, United Kingdom (Age: *M* = 26.00 years; *SD* = 5.17).^2^ The Chinese monolingual speakers were selected from a senior vocational high school in Yantai, China (Age: *M* = 19.30 years; *SD* = 0.97). It seems that they may not match exactly with other groups in aspects such as age and educational background. This is because it is virtually impossible to recruit entirely and completely monolingual Chinese native speakers who are also educated to university level. Efforts have been made to ensure that the technical school students recruited have only basic to lower knowledge of English due to the course design in their school.

In addition to the two monolingual groups, three groups of Chinese learners of English were recruited from a university in Shenzhen, China. These L2 learners were at three proficiency levels: elementary, intermediate and advanced. Their proficiency levels were determined by their test scores in the English Language Proficiency Tests, administered twice a year by the Ministry of Education, China. As the official measure of English proficiency, these tests distinguish three tiers: Band 4, Band 6, and Band 8. Separate test papers were designed for the three bands, in listening, reading, writing, comprehension and translation, with identical methods of marking but different proficiency requirements. People who had passed Band 4 with no other tests indexing a higher level of English proficiency were classed as beginners (Age: *M* = 20.28, *SD* = 1.76; Test score: *M* = 70.97 [out of 100], *SD* = 6.32; L2 exposure = 7.19 years). L2 learners who passed Band 6 were classified as intermediate proficiency (Age: *M* = 21.16, *SD* = 1.14; Test score: *M* = 69.23 [out of 100], *SD* = 6.23; L2 exposure = 8.06 years); those who had passed Band 8 were classed as advanced L2 learners (Age: *M* = 24.77, *SD* = 2.06; Test score: *M* = 70.89 [out of 100], *SD* = 6.70; L2 exposure = 11.61 years).

All L2 learners had taken these English proficiency tests 6 months or so prior to the experiment. They all started their English learning at around the age of 10, in a predominately Chinese–speaking environment, in which their English input was mainly from classroom instruction.

### Materials

The experimental stimuli consisted of 16 triads of video clips (5 s each) demonstrating spontaneous motion events (Ji, 2018). They all depicted a boy named Bonny performing a specific action (e.g., walking, running, hopping) along certain route (e.g., vertical: *up, down*; boundary–crossing: *into, across*; deixis: *toward, away from* and course parallel to the Ground of motion: *along, around*). In each triad, there were three video clips: a target and two alternates. Apart from the motion itself, these videos were identical in aspects such as the background scenery for motion and the protagonist’s clothing in order to direct the participants’ attention to actions rather than anything else and to help them understand that the judgments need to be made on the basis of similarities in actions.

In each triad, the video clips were played in a synchronized sequence. The target video was played first, in the central position of a black screen, for 5 s. After 0.5 s of a totally black screen, the two alternate videos appeared side–by–side on the same screen for 5 s. There was a 1 s black screen between triads. Compared with the target video clip, which incorporated both a Manner and a Path (e.g., Bonny hopped out of the bedroom), the Manner–matched alternate changed the trajectory of motion whilst keeping the Manner of motion intact (e.g., Bonny hopped *into* the bedroom) while the Path–matched alternate retained the Path of motion but altered the Manner of motion (e.g., Bonny *limped* into the bedroom). Appendix A shows a complete list of the 16 target and alternate actions and Appendix B gives an example of the video stimuli used.

Audio instructions accompanied the video stimuli. Each video clip was labeled with a number and no descriptive language was used in the audio instruction. For instance, the participants heard on one occasion, “This is 5.” during the target, and “Which one is most like 5?” when the target finished and the alternates began. The task is therefore non-linguistic. Note that the audio instructions were given in English, but the language used to complete prior-to-experiment consent forms and demographic information sheets was Chinese. According to some previous findings, the language involved in a non-verbal task may exert a contextbound and transient effect on bilingual cognition, but if bilingual participants can access two languages during the experiment, then possible effects induced by language context may be wiped out (e.g., Athanasopoulos et al., 2015; Montero-Melis et al., 2016, pp. 640–642).

### Procedures

The experiments were conducted in different locations in London and in China. Each location consisted of a quiet classroom (or seminar room) with no (or little) distraction for participants. Informed consent forms and demographic information sheets (e.g., age, course of study, parental education, language exposure) were gleaned from participants before the experiment. The study was approved by the Academic Committee of the university where the author works. Each participant received a participation fee of reasonable amount upon completing the task.

Prior to the testing session, a perseveration test was administered with the aim of eliminating any possible ‘lateral order effects.’ Participants were shown five triads of pictures with the target picture showing an ordinary object (e.g., a small blue bird) and two alternates differing from the target in size only (e.g., a *big* blue bird) or in color only (e.g., a small *yellow* bird). The alternates were arranged side-by-side on the same page. Anyone who chose alternates from only one side of the page for all triads (none did actually) was considered perseverative and therefore, excluded from the testing phase.

The participants took part in the test individually. They were invited to view video clips played on a MacBook Pro and requested to judge the similarity between motion scenes by pressing one of the two keys on the keyboard: ‘A’ or ‘L’, respectively. These two keys were kept apart from each other on the keyboard and covered with white stickers (i.e., no linguistic labeling). If the participant felt that the alternate on the left side of the screen was most like the target, she should press down ‘A’; otherwise, she chose the ‘L’ key.

There was a training triad prior to the test session (target: *The boy pulling a boat out of lake*), which aimed to familiarize the participants with test procedures and requirements. Participants were encouraged to make their decisions as quickly as possible. As soon as the training phase ended and the testing session started, the female experimenter removed herself from the participant’s view by retreating to a far corner of the room. All video stimuli were played to the participants on the laptop screen through the stimulus presentation software ‘SuperLab 4.5.’ At the end of each session, a file was automatically generated by the software, which contained, among other things, participants’ choices (‘A’ or ‘L’ key pressed) and the time they spent in making that choice (in milliseconds).

The stimuli were played in two randomized orders: A and B, which were counterbalanced across participants in a given group. The presentation position of Manner– or Path–matched videos (left or right side of the screen) was also counterbalanced in a given order. In addition, in order to prevent participants from subconsciously verbalizing the motion events, a ‘number-shadowing’ condition was provided in which numbers in random sequence were ‘shadowed’ (i.e., broadcasted aloud) to participants, aiming to elicit ‘non-linguistic’ thinking. The participants heard the same list of random numbers throughout the testing session and they were not required to repeat the numbers aloud.

### Coding

Two sets of variables were adopted in data coding. The first type of measurement is categorical in nature and refers to participants’ overt choices in decision making, as indicated by their pressing down of given keys (‘A’ or ‘L’) on the keyboard (i.e., either Manner–match or Path–match). This categorical variable aims to reveal whether there are differences across groups in terms of preference.

The other type of variable is continuous in nature and aims to test the degree of differences (if any) between groups. It refers to participants’ latencies in response to different non-linguistic stimuli, i.e., RT. According to some previous studies (e.g., Hunt and Banaji, 1988; Hunt and Agnoli, 1991), differences in non-linguistic cognition engendered by language differences will be more obvious in implicit processing or in speed of processing than in ‘A or B’ responses to classification tasks. This is because the processing variable enables us to judge the degree of difference rather than its presence or absence. Although few crosslinguistic studies have utilized RT measurements in behavioral tasks, Hunt and Banaji (1988) found that speakers of different languages spent subtly different amounts of processing time in judging the same situation, presumably due to their different ways of linguistically encoding the situation. In this light, the data coding in the present study combines overt preferences with implicit processing, with the aim of more precisely revealing any potential differences in non-linguistic thought patterns produced by linguistic differences.

In our study, the participants were given an instruction sheet immediately prior to the experiment. The instructions clearly requested that participants ‘make their choices as quickly as possible.’ The RT for a given stimulus was calculated from the onset of alternate videos to the completion of a given trial, including a 1 s black screen immediately following the end of alternate videos. Theoretically, the longest RT could be 6,000 ms. However, the participants were told that they did not have to wait until they had seen alternate videos in their entirety. By excluding extremely short values (button pressed within 200 ms of stimulus onset), 93 out of 2659 values (3.50%) were removed from the RT data. A prior screening for outliers was conducted by removing all observations that were at a distance of more than two standard deviations (SD) from the group mean for long RTs, and 71 observations out of a total of 2559 (2.77%) were cleaned. In addition, 7 observations (0.27%) were lost due to technical failure, leaving 2488 observations (97.23%) for final analysis.

### Data Analysis

We used R and lme4 (Bates et al., 2012) to perform two linear mixed effects analyses on the dependent variables: (i) the decision strategy (i.e., preferences); (ii) the RT. We used linear mixed-effects (LME) modeling via restricted maximum likelihood for repeated-measures analyses (Judd et al., 2012). As random effect, we had intercepts representing participant number and stimuli items. For the preferences, participants groups (i.e., five levels) were entered as a fixed-effect factor, i.e., preference ∼ group + (1|particpant) + (1|stimuli). For the RTs, participant groups (i.e., five levels) and preferences (i.e., path- vs. manner- match) were treated as two fixed-effect factors, i.e., RT ∼ group^∗^preference + (1|particpant) + (1|stimuli). To assess the validity of the mixed effects analyses, we performed likelihood ratio tests comparing the models with fixed effects to the null models with only the random effects. We rejected results in which the model including fixed effects did not differ significantly from the null model.

## Results

Using both categorical choices and continuous measurements, this section investigates: (a) whether, and how, monolingual speakers’ decision strategies differ significantly from that of L2 learners; (b) whether, and how, typological variations in motion description lead to behavioral differences between Chinese and English monolingual speakers; c. whether, and how, the behavior of L2 learners differs as a result of their progression in language proficiency^3^.

### Mean Number of Manner–Matches and Path–Matches Across Five Participant Groups

**FIGURE 1:**
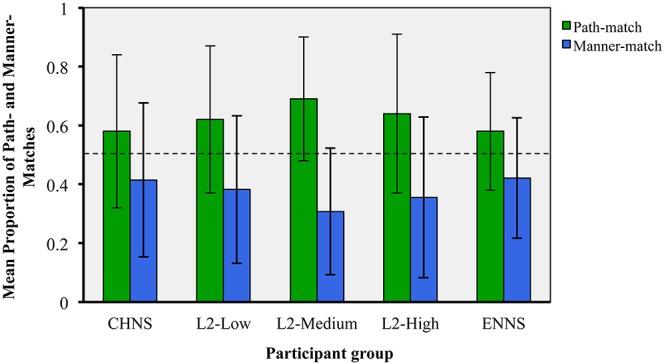
Mean proportions of the Path–match and Manner-match across participant groups (error bars represent *SD*s).

The overt choices of participants were determined by the given key (A or L covered with a white sticker) that they pressed down during the experiment. All choices were sorted into two categories: Manner–match and Path–match. Figure 1, below, represents the mean proportion of Manner– and Path–matches across participant groups.

A visual inspection of Figure 1 seems to suggest that the monolingual speakers of both English and Chinese, as well as the L2 learners of English, prefer the Path–match over the Manner–match. To verify this observation, we investigated if the proportion of preferences for the Path-match significantly differed from the chance level (50%; note that the proportion of the Path-match is complementary with that for the Manner–match). We conducted one-sample *t*-tests (against 0.5) for each participant group. The results revealed that the proportion for the Path-match was significantly above the chance level among groups of ENNS [*M* = 0.58; *SD* = 0.20; *t*(31) = 2.10, *p* < 0.05], L2-Low [*M* = 0.62; *SD* = 0.25; *t*(31) = 2.60, *p* < 0.05], L2-Medium [*M* = 0.69; *SD* = 0.21; *t*(31) = 5.04, *p* < 0.001] and L2-High [*M* = 0.64; *SD* = 0.27; *t*(31) = 2.95, *p* < 0.01]. In addition, the proportion of Path-matched preferences was marginally significantly above the chance level for the group of CHNS [*M* = 0.58; *SD* = 0.26; *t*(31) = 1.82, *p* = 0.07]^4^.

Further, we examined if the preferences differed significantly across the five participant groups (CHNS, L2–Low, L2–Medium, L2–High and ENNS). Mixed models analysis was carried out using R (version 3.0.3) with glmer function and the package lmerTest to obtain parameter estimates. The overt choice of participants was coded as a binomial dependent variable: the preference for the Path-match was coded as “1” and that for the Manner-match was coded as “0”. It was found that the main effect of participant group was not significant, χ*^2^*(4) = 5.17, *p* > 0.1, thus suggesting that participants, irrespective of their language group (i.e., English vs. Chinese), learner type (i.e., L1. Vs. L2 learners) and proficiency level (i.e., low to high), showed a shard tendency for the Path-match in their choices.

We also used R and lme4 to perform a further linear mixed effects analysis with participant group (CHNS, L2-Low, L2-Medium, L2-High, ENNS) as between subjects factors and test item (16) as a within-subjects factor, i.e., preference ∼ group^∗^item + (1|participant). To assess the validity of the mixed effects analyses, we performed likelihood ratio tests comparing the models with fixed effects to the null models with only the random effects. We rejected results in which the model including fixed effects did not differ significantly from the null model. It was found that there was a significant difference across test items (χ^2^ = 348.96, df = 15, *p* = 0.001), suggesting that a particular item was viewed as more salient in Manner or in Path.

To illustrate, the frequency of the Path–match with item 6 (target: crawling into the cave; alternates: crawling up the cave and jumping into the cave) fell below the chance level (50%; i.e., more Manner–matches): *t*(159) = −3.607, *p* < 0.001 (*M* = 0.36, *SD* = 0.482), whereas the frequency of the Path–match with item 11 (target: walking toward the house; alternates: walking *out of* the house and *jogging* toward the house) was significantly above the chance level [i.e., more Path–matches, *t*(159) = 16.813, *p* < 0.001 (*M* = 0.900, *SD* = 0.301)]. Such findings suggest that components of the spontaneous motion events as illustrated in certain test items are more Manner–match eliciting or more Path–match eliciting.

### RT in Judgment Amongst Five Participant Groups

**FIGURE 2:**
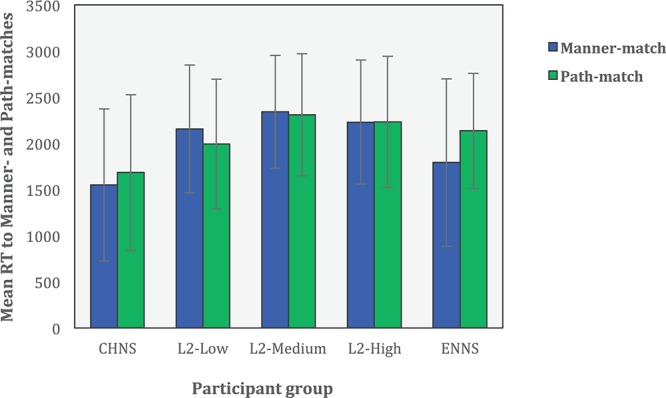
Mean RT (in millisecond) to Manner– and Path–matches across participant groups (error bars represent *SD*s).

Apart from overt choices, the response latency of participants in their judgments was examined using the continuous measurement of RT in milliseconds (see Figure 2 and Table 1).

**TABLE 1:** Mean RT (in millisecond) to spontaneous motion scenes in 5 participant groups.

**Group**	**Mean overall RT (*SD*)**	**Mean RT to Manner–match (*SD*)**	**Mean RT to Path–match (*SD*)**
CHNS	1466 (*986*)	1434 (*948*)	1489 (*1013*)
L2–Low	2300 (*910*)	2402 (*892*)	2234 (*916*)
L2–Medium	2575 (*827*)	2527 (*764*)	2596 (*854*)
L2–High	2591 (*934*)	2601 (*929*)	2586 (*938*)
ENNS	2156 (*1035*)	1844 (*1180*)	2388 (*844*)

RT to Manner-match: CHNS < ENNS, L2-Low, L2-Medium, L2-High, *ps* < 0.001; ENNS < L2-Low, L2-Medium, L2-High, *ps* < 0.001.

RT to Path-match: CHNS < ENNS, L2-Low, L2-Medium, L2-High, *ps* < 0.001; L2-Low < L2-High, *p* < 0.05.

Mixed models analysis was carried out using R with lmer function in the package lmerTest to obtain parameter estimates. The model includes participant group (CHNS, L2–Low, L2–Medium, L2–High and ENNS) and preference type (i.e., Manner–match and Path–match) as fixed effects, with participants and items as random effects. The RT was coded as the dependent variable. The results of the analyses were reported as follows.

There was a significant main effect of preference, χ*^2^* (1) = 26.57, *p* = <0.001, with participants responding slower in the Path-match (*M* = 2271 ms, *SD* = 999) condition than in the Manner-match condition (M = 2114 ms, *SD* = 1066), b = 205.92, *SE* = 39.65, *t* = 5.193, *p* = <0.001. The main effect of participant group was also significant, χ*^2^* (4) = 78.12, *p* < 0.001. More importantly, these two main effects were qualified by a significant interaction between participant group and preference, χ*^2^*(4) = 43.99, *p* < 0.001. We proceeded to compare the RT to the Path-match with that to the Manner-match in each participant group. Further analyses showed that the participants in the ENNS group were significantly slower in the Path-match condition than in the Manner-match condition, *b* = 613.79, *SE* = 92.22, *t* = 6.66, *p* < 0.001. The other group of monolingual speakers (i.e., CHNS), as well as the three L2 groups, did not differ significantly in their RTs to the Manner- and Path-matches, all *p*s > 0.1.

Further, we decomposed the interaction effect from the other direction, looking at group differences in RTs in Path- vs. Manner-match conditions, respectively. For the Manner-match condition, participants in the CHNS group responded significantly faster than those in the other monolingual group (i.e., ENNS; *b* = 405.6, *SE* = 139.8, *t* = 2.90, p < 0.01) and the three groups of L2 learners (vs. L2-Low, *b* = 929.9, *SE* = 142.9, *t* = 6.51, *p* < 0.001; vs. L2-Medium, *b* = 1099.8, *SE* = 147.4, *t* = 7.46, *p* < 0.001; vs. L2-High, *b* = 1180.5, *SE* = 146.8, *t* = 8.04, *p* < 0.001). Also, the RT was significantly shorter in the ENNS group than in all three L2 groups (vs. L2-Low, *b* = 524.3, *SE* = 141.9, *t* = 3.70, *p* < 0.001; vs. L2-Medium, *b* = 694.2, *SE* = 146.4, *t* = 4.74, *p* < 0.001; vs. L2-High, *b* = 774.8, *SE* = 145.7, *t* = 5.32, *p* < 0.001). No other significant differences were detected between any other two groups.

For the Path-matched condition, participants in the group of CHNS responded faster as compared to those in all the other four groups (vs. ENNS, *b* = 982.7, *SE* = 153.0, *t* = 6.43, *p* < 0.001; vs. L2-Low, *b* = 835.1, *SE* = 153.4, *t* = 5.45, *p* < 0.001; vs. L2-Medium, *b* = 1108.0, *SE* = 152.1, *t* = 7.29, *p* < 0.001; vs. L2-High, *b* = 1173.9, *SE* = 153.1, *t* = 7.67, *p* < 0.001). Amongst L2 learners, the low-proficiency participants reacted significantly quicker than advanced learners (*b* = 338.8, *SE* = 152.7, *t* = 2.22, *p* < 0.05). No other significant differences were reported between any other two groups.

## Discussion and Conclusion

The present investigation examines whether, and how, subtle linguistic differences in grammar (as demonstrated in a lexicalization strategy of motion events) have a mind–shaping effect on the motion event cognition of Chinese–English bilinguals, compared with monolingual speakers. Two sets of data were collected, regarding selection behavior and implicit processing time, respectively. Our major findings are twofold. First of all, as regards monolingual speakers, the Chinese participants spent an approximately equal amount of time in making Path- and Manner-matched judgments whilst their English counterparts used much longer time in judging Path similarities than in evaluating Manner resemblance between motion scenes. The overall quicker response of Chinese participants was attributable to their quicker reaction to both Path-matched and Manner-matched scenes, as compared to the English monolinguals.

Secondly, the L2 learners, even at the advanced stage of acquisition, resembled the source language (Chinese) speakers in allotting a roughly equal amount of time in making Manner- and Path-matched responses. Meanwhile, they did not achieve the behavioral efficiency in judging Manner similarities as compared to the target language (English) speakers. In addition, the L2 learners were generally slower in response than their monolingual peers. Virtually, no major developmental changes in the pattern of response latency, particularly in terms of RT in Manner-matches, were attested across proficiencies in L2 learners.

Several observations from our findings deserve a closer examination. First of all, our participants demonstrate a shared orientation toward Path-matches in their overt selections. Such results can be interpreted in quite different ways. It is possible that great variations between languages in motion description tend to be superficial, non-categorical and probabilistic, and do not go beyond the level of language performance to penetrate into the cognitive domain. In other words, linguistic differences in motion description can be regarded, in some sense, as varied instantiations of a common underlying conceptual framework. In light of Talmy’s (1985) Path salience hypothesis, Path (rather than Manner) is the most central and indispensable ingredient for any motion event. This may explain why in terms of selection strategy, the participants in our study opt for Path-matches most frequently.

Despite this shared tendency toward the Path dimension, our analysis of RT reveals significant differences between monolingual speakers of different languages, as well as differences between monolingual vs. bilingual speakers. Taking into account these observations, it seems more likely that the effect of language typology does exist but fails to be brought forth due to various factors. For instance, the typological distance between equipollently framed Chinese and satellite-framed English may be too close to allow for any difference in behavioral pattern to surface. In this case, future studies involving an additional verb-framed language, such as Spanish or French, may illustrate the issue more clearly. Also, the measurement of categorical preferences might be too ‘coarse’ to bring forth any variation in behavioral pattern resulting from minimal differences between languages in motion description. Seen this way, the finer and more subtle measure at the automatic implicit level of conceptualization (i.e., RT) help reveal a degree of differences in thought pattern that has remained elusive under the categorical gauge.

In addition, there is a third likelihood that the potential language effect has been canceled or nullified by the verbal interference task utilized in the non-linguistic experiment. This possibility is in line with the notion that speakers of satellite-framed languages are more likely than those of verb-framed languages to base their similarity judgments on the Manner dimension in the experimental situation, as found in previous studies. However, when the recruitment of languages was hindered through verbal interference, crosslinguistic differences disappear (Montero-Melis and Bylund, 2016; see also Athanasopoulos et al., 2015)^5^.

Equally possibly, the lack of effects of motion event typology has something to do with the specific way the video stimuli are played to the participants in our particular task (i.e., simultaneously rather than one after the other). Some previous studies (Finkbeiner et al., 2002; Athanasopoulos and Bylund, 2013) suggested that whether or not the two alternates in a triads matching task are shown simultaneously has an impact on the presence or absence of language effects. To cite an example, Finkbeiner et al. (2002) reported that when alternates in a triad were played one after the other, participants tended to show different preferences that can be mapped onto language differences. Such effects, however, were found to disappear when alternates were played to the participants simultaneously (p. 18). They argued that the reason for the surfacing of language effects is the component of working memory, which has encouraged the subconscious use of language in judgment when the alternates are played sequentially. Seen in this way, the methodological decision of playing stimuli simultaneously, following Finkbeiner et al. (2002), may have minimized the cross-language effects in this study.

The second issue meriting further exploration is the significantly shorter RT of Chinese monolingual speakers compared with their English counterparts. A closer look at the data further reveals that their overall quicker response time can be attributed to their particularly higher efficiency in both Manner– and Path–matched judgments. These findings are consistent with those reported in Ji and Hohenstein (2017), who investigate the L1 conceptualization of similar motion events in English and Chinese children across ages, as well as adults. They suggest that this phenomenon can be approached from varied perspectives, such as decision strategy (e.g., ‘jump–to–conclusion’ in Chinese monolinguals vs. ‘wait–and–see’ in English monolinguals) and culture–specific viewpoints (a ‘holistic’ perspective of events in eastern culture vs. ‘analytical’ perspective of events in western culture). Amongst various possible explanations, it is argued that the attested language effect may be better illustrated in terms of an information processing mode. Specifically, Chinese grammar, through a verb compound in lexicalization pattern, highlights both Manner and Path in its linguistic encoding of motion events. This particular aspect of the Chinese language prompts its speakers to direct an equal amount of habitual attention to these two semantic components for motion, which fosters a ‘Manner–and–Path salience’ cognitive pattern. Therefore, at the processing level, Chinese speakers tend to simultaneously assess the similarity in Manner and Path dimensions in a ‘parallel’ fashion. In comparison, the English language, in the most marked grammatical category of main verb, encodes Manner only. English speakers therefore tend to direct their habitual attention to Manner first and develop a conceptualization pattern in which Manner is given greater prominence, eventually leading to their ‘sequential’ mode of information processing: the similarity in Manner is evaluated in the first instance, followed by an assessment along the Path dimension. A final selection is made only after semantic components for motion are weighed one by one, which lengthens the RT on the part of English monolinguals (see Ji and Hohenstein, 2017, pp. 67–68 for a more detailed discussion; see also Rousselet et al., 2010).

In other words, Path is an attribute prominently marked in Chinese but not in English, whereas Manner is prominently marked in both languages. This means that Chinese monolinguals, in contrast with their English counterparts, attend more strongly to Path of motion, develop a cognitive pattern in which Path is given greater prominence than in English, and respond more quickly to the Path attribute of motion events in categorization or judgment (see similar findings in Kersten et al., 2010). This explains, at least partly, why the RT to the Path-match is exceptionally significantly shorter in Chinese than in English monolinguals.

The overall RT of the three groups of L2 learners is significantly slower than monolingual speakers of both languages. In particular, they are less efficient in judging Manner-matched scenes compared with the speakers of the target language (i.e., English monolinguals), even at the advanced stage of acquisition. As proposed by Pavlenko (2011), learning an additional language involves a process of conceptual adjustment or switch, for instance, a process of converging L1 and L2 categories or perspectives. Results, as such, seem to suggest that a shift in perspective, for instance shifting from a conceptual pattern in which Manner and Path have equal prominence (i.e., Chinese) to a cognitive mode in which Manner has a greater prominence (i.e., English) can be both cognitively demanding and time-consuming.

Our findings for L2 learners reveal that the bilinguals in this study are not comparable to their monolingual counterparts in terms of response latency. Such a phenomenon can be approached, in the first instance, by taking into consideration some (extra)linguistic factors. Pavlenko (2011) proposes several variables contributing to the attainment of an L2 learner in conceptual restructuring, which involve the age of L2 acquisition, the context of acquisition, the length of exposure to the target language, language proficiency and the frequency of target language use (2011: 249–251). In this light, the Chinese-English bilinguals in our study are largely disadvantaged. To illustrate, these Chinese learners started their English learning around the age of 10–12. As reported by Hohenstein et al. (2006), speakers who acquire their L2 before the age of 5 are more likely to display target-like responses in linguistic and behavioral tasks than those who start L2 learning at a later stage of acquisition (e.g., after 12 years old). In terms of the language-learning context, the L2 learners in the present study live in a predominately Chinese–speaking environment where they use only Chinese in their daily life. Their English input is rather limited and mainly comes from classroom teaching, which averages about four hours per week. Previous research has revealed that immersion in an L2 context facilitates conceptual restructuring; years of immersion create a more reliable predictor of target-like behavior than years of formal instruction (see Pavlenko, 2011: 250 for a summary of relevant findings). None of the bilinguals in our study, however, had the experience of visiting or living in an English-speaking country for a period of longer than two weeks. Furthermore, they used English at a very low frequency and mainly in English classroom activities. Seen this way, it is understandable that the RT evidence in the present study suggests that these bilinguals are in a stage of on-going restructuring of their conceptual frameworks.

Further, some previous studies in bilingual cognitive development once claim that bilingualism can extend one’s cognitive capacities and bilingual speakers can outperform their monolingual peers in nonverbal cognitive tasks involving control processes, such as selective attention to given aspects of a problem and switching between competing alternatives (Kharkhurin, 2010: 213; see also Bialystok, 2009). However, such an effect of bilingualism on an individual’s executive control functioning has been recently challenged. A growing number of studies suggest a lack of robustness and reliability of such a bilingualism effect with some researchers even discrediting the existence of bilingual cognitive advantages per se (see, for instance, Paap and Greenberg, 2013; Paap et al., 2015; Branzi et al., 2016; von Bastian et al., 2016). This, coupled with some particular disadvantages of our participants (a late age of acquisition, restricted L2 context, etc.), leads to the largely target-deviant pattern of RT in L2 learners across proficiencies.

Our research findings are generally in line with the results from investigations of L2 linguistic encoding of motion events, particularly those involving an acquisition of advanced linguistic skills, such as syntactic organization and discourse strategy. To cite an example, Ji and Hohenstein (2014a, b) systematically investigate how English learners of Chinese syntactically package varied semantic components for complex caused motion events (e.g., Manner, Cause, Path, etc.). It is reported that even the advanced L2 learners have not fully acquired the typical syntactic pattern in motion description in the target language. They have arrived, instead, at an inter–language, showing some resemblance to the target system as well as some traces of L1 influence. Furthermore, no developmental tendency was observed at the initial and intermediate stages of acquisition; non-significant changes occurred only when learners progressed to an advanced level. Taken together, findings in L2 motion representation, at both linguistic and conceptual levels, suggest a general difficulty in fully adapting to the target pattern in L2 learners. Seen in this way, these results seem to be consistent with a weak version of the linguistic relativity hypothesis. In Slobin’s thinking for speaking hypothesis, he argues that a native language “is not a neutral coding system of an objective reality,” but instead is a system that has trained its speakers from early on to pay attention to specific aspects of events and experience when talking about them (Slobin, 1996, p. 89). ‘Thinking for speaking’ involves picking those characteristics of objects and events that simultaneously fit some conceptualization of the event and are readily encodable in the language (Slobin, 1996, pp. 75–76). In this sense, one’s thinking for speaking pattern, which is ingrained from the childhood, should be particularly resistant to remolding in adult L2 acquisition.

The linguistic and conceptual representation of motion events in the L2 domain is a complex issue, involving multiple factors. These include, but are not restricted to, dimensions such as the directionality of language (and conceptual) transfer, nature of motion stimuli used (i.e., spontaneous vs. caused motion), type of measurement adopted (i.e., explicit choices vs. implicit processing speed) and task–specific requirements (e.g., memory recognition vs. categorization). In general, findings from previous investigations seem varied. On the one hand, much research detected the effects of L1 on linguistic expression and on the conceptualization of motion events in L2 learners, particularly in those of low proficiency. On the other, behavioral and neurophysiological evidence reveals that human linguistic and cognitive systems can be highly adaptive and flexible. Some key questions need to be addressed in future studies in the domain of bilingual cognition of motion events. These include: which aspect(s) of language (e.g., grammatical aspect, lexicalization) affects which part of the cognitive system in L2 learners (e.g., sensory perception, object categorization)? What is the exact mechanism that determines the extent and nature of cognitive restructuring in L2 learners? What is the relative weight of varied linguistic and sociocultural factors that modulate the effect of bilingualism on cognition?

## Data Availability Statement

All datasets generated for this study are included in the article/Supplementary Material.

## Ethics Statement

The studies involving human participants were reviewed and approved by Academic committee for humanities and social sciences, Shenzhen University. The patients/participants provided their written informed consent to participate in this study.

## Author Contributions

The author confirms being the sole contributor of this work and has approved it for publication.

## Conflict of Interest

The authors declare that the research was conducted in the absence of any commercial or financial relationships that could be construed as a potential conflict of interest.

## Footnotes

1 Abbreviations used in Section “Motion Event Typology and Its Cognitive Implications” are as follows: C, Component; DUR, Durative aspectual marker *zhe* in Chinese.

2 ‘M’ here stands for ‘Mean’ and ‘SD’ represents ‘standard deviation’.

3 Replication data for all the results reported in this section are available via / 10.18710/N8KO4O (Ji, 2018).

4 The abbreviations for participant groups are as follows: CHNS, Chinese monolingual speakers; ENNS, English monolingual speakers; L2_Low, L2 learners of English with low proficiency; L2_Medium, L2 learners of English with intermediate proficiency; L2_High, L2 learners of English with high proficiency.

5 Note that in our study, the hindrance in language recruitment only eliminated differences in choice judgment but not in RT pattern. It is presumed that this might reflect the contrast between attentional allocation at the explicit level and information processing mode at the implicit level. Specifically, without the ‘marking’ function of language, the habitual attention of participants was no longer directed to given aspects of events that have been made readily encodable in their language (i.e., Manner in English vs. Manner and Path in Chinese). Rather, they all attended to the most basic dimension of motion (i.e., Path) in choice judgment. However, at the implicit level, the routine of information processing was not altered. Chinese monolinguals may process Manner and Path at the same time whilst their English peers may process Manner information in the first instance (see a discussion of ‘parallel’ vs. ‘sequential’ processing mode on p. 25).
