# Supplementary material for: Cognitive Representation of Spontaneous Motion in a Second Language: An Exploration of Chinese Learners of English
Source: Front Psychol. 2019 Dec 3;10:2706. doi: 10.3389/fpsyg.2019.02706 (PMC6902645; doi:10.3389/fpsyg.2019.02706)
Supplement: Supplementary file 1 [file Data_Sheet_1.docx]

**Appendix A** Description of 16 triads of motion stimuli in the experiment

1. a. He walked down the stairs.

b. He walked *up* the stairs.

c. He *jumped* down the stairs.

2. a. He tiptoed down the roof of the barn.

b. He tiptoed *across* the roof of the barn.

c. He *hopped* down the roof of the barn.

3. a. He drove a car up the hill.

b. He drove a car *towards* the hill.

c. He *rode* a horse up the hill.

4. a. He skipped down the bank.

b. He skipped *along* the bank.

c. He *slid* down the bank.

5. a. He hopped out of the room.

b. He hopped *into* the room.

c. He *limped* out of the room.

6. a. He crawled into the cave.

b. He crawled *up* the cave.

c. He *jumped* into the cave.

7. a. He ran into the post office.

b. He ran *away from* the post office.

c. He *limped* into the post office.

8. a. He skateboarded out of a big house.

b. He skateboarded *along* a row of houses.

c. He *walked on stilts* out of a big house.

9. a. He sauntered away from the fountain.

b. He sauntered *towards* the fountain.

c. He *hopped* away from the fountain.

10. a. He ran away from the icy slope.

b. He ran *down* the icy slope.

c. He *jumped* away from the icy slope.

11. a. He walked towards the house.

b. He walked *out of* the house.

c. He *jogged* towards the house.

12. a. He skated away from the ice sculpture.

b. He skated *around* the ice sculpture.

c. He *sledged* away from ice sculpture.

13. a. He jumped around a flower stand.

b. He jumped *along* a row of flower stands.

c. He *crawled* around a flower stand.

14. a. He jumped along a row of benches.

b. He jumped *down* a bench.

c. He *waddled* along a row of benches.

15. a. He walked on stilts around the tennis court.

b. He walked on stilts *across* the tennis court.

c. He *skateboarded* around the tennis court.

16. a. He hopped along a row of bushes.

b. He hopped *towards* the bushes.

c. He *ran backwards* along a row of bushes.
