# Supplementary material for: Cognitive Representation of Spontaneous Motion in a Second Language: An Exploration of Chinese Learners of English
Source: Front Psychol. 2019 Dec 3;10:2706. doi: 10.3389/fpsyg.2019.02706 (PMC6902645; doi:10.3389/fpsyg.2019.02706)
Supplement: Supplementary file 2 [file Data_Sheet_2.docx]

**Appendix B** An illustration of the video stimuli showing spontaneous motion events

**
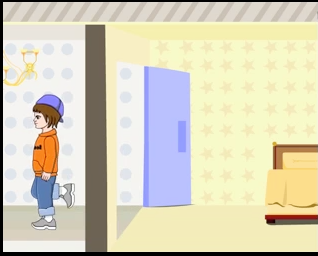
**

5a. Bonny hopping out of bedroom


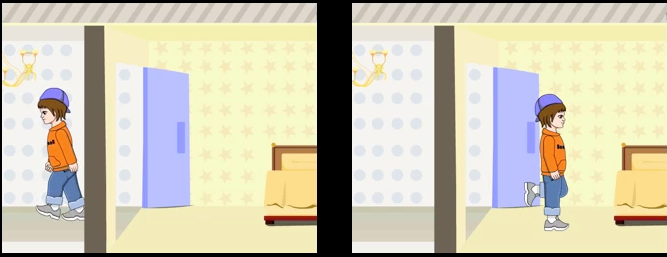


5b. Bonny LIMPING out of bedroom 5c. Bonny hopping INTO bedroom

Audio instructions accompanying the videos were:

Target: “This is 5”.

Alternates: “Which one is most like 5?”
